# Endoplasmic reticulum stress: a novel targeted approach to repair bone defects by regulating osteogenesis and angiogenesis

**DOI:** 10.1186/s12967-023-04328-8

**Published:** 2023-07-18

**Authors:** Tingyu Wu, Yaping Jiang, Weipeng Shi, Yingzhen Wang, Tao Li

**Affiliations:** 1 Department of Joint Surgery, The Affiliated Hospital of Qingdao University, No. 59, Haier Road, Qingdao, 266003 China; 2 Department of Oral Implantology, The Affiliated Hospital of Qingdao University, Qingdao, 266003 China

**Keywords:** Endoplasmic reticulum stress, Unfolded protein response, Osteogenesis, Angiogenesis, High glucose, Inflammation, Exosome, Bone defects

## Abstract

Bone regeneration therapy is clinically important, and targeted regulation of endoplasmic reticulum (ER) stress is important in regenerative medicine. The processing of proteins in the ER controls cell fate. The accumulation of misfolded and unfolded proteins occurs in pathological states, triggering ER stress. ER stress restores homeostasis through three main mechanisms, including protein kinase-R-like ER kinase (PERK), inositol-requiring enzyme 1ɑ (IRE1ɑ) and activating transcription factor 6 (ATF6), collectively known as the unfolded protein response (UPR). However, the UPR has both adaptive and apoptotic effects. Modulation of ER stress has therapeutic potential for numerous diseases. Repair of bone defects involves both angiogenesis and bone regeneration. Here, we review the effects of ER stress on osteogenesis and angiogenesis, with emphasis on ER stress under high glucose (HG) and inflammatory conditions, and the use of ER stress inducers or inhibitors to regulate osteogenesis and angiogenesis. In addition, we highlight the ability for exosomes to regulate ER stress. Recent advances in the regulation of ER stress mediated osteogenesis and angiogenesis suggest novel therapeutic options for bone defects.

## Introduction

As the elderly population has increased globally, so has the number of patients with clinical bone defects [1, 2]. Patients with diabetes mellitus have a high incidence of bone defects [3]. Diabetes mellitus impairs bone regeneration and biomechanics in newly regenerated bone, which may be related to metabolic disorders and dysfunction of mitochondrial function and macrophage polarization induced by blood glucose fluctuations, leading to the production of reactive oxygen species (ROS), which creates an inflammatory microenvironment at the site of bone defect [4, 5]. At present, in the field of bone tissue engineering, increasing studies indicate that the functional polarization of macrophages can be adjusted by various modified hydrogels and 3D bioprinting of multicell-laden scaffolds, so as to promote the repair of diabetic bone defects [6, 7]. The latest research shows that stem cell therapy can also be a therapeutic target, bone marrow-derived macrophage (BMDM) -derived exosomal miRNA can affect bone marrow mesenchymal stem cell (BMSCs) differentiation, providing effective methods and potential therapeutic targets for the treatment of diabetic bone defects [8]. However, the role of BMD-derived exosomal miRNA in diabetes and their communication with BMSCs remains unknown. The specific mechanisms underlying impaired bone repair and regeneration in diabetic conditions remain to be investigated.

Bone regeneration requires both osteogenesis and angiogenesis [9]. The mechanisms of bone regeneration include membrane-internalized bone and cartilage-internalized bone [10]. Bone regeneration requires the participation of osteoblasts, osteoclasts, and chondrocytes. Endothelial cells (ECs) promote angiogenesis, thereby contributing to bone regeneration [11]– [13]. Angiogenesis depends on the coordination of pro- and anti-angiogenic factors [14]. Vascular endothelial growth factor (VEGF) and fibroblast growth factor (FGF) were the earliest identified pro-angiogenic factors [15]. They drive EC proliferation, migration, and differentiation to promote angiogenesis [16].

The cellular and molecular mechanisms of angiogenesis and osteogenesis in bone regeneration have been investigated. However, ER stress pathway has received little attention. ER stress is related to many human diseases [17]. Drugs targeting ER stress have been developed [18]. ER stress is a double-edged sword that determines whether cells survive or die [19]. Appropriate ER stress restores cellular homeostasis by activating adaptive cellular adaptive programs, whereas excessive ER stress induces cell death by triggering apoptosis [20].

ER stress has dual roles in the regulation of osteogenesis and angiogenesis. Here, we review the effect of ER stress on osteogenesis and angiogenesis, including the link between HG, inflammation and ER stress signaling pathways. As a subclass of extracellular vesicles, exosomes come from a wide range of sources, can be secreted by almost all kinds of cells, and exist in various body fluids [21]. ER stress can promote exosome formation and release [22–24]. We also reviewed that exosomes from different sources promote osteogenesis and angiogenesis. Therefore, it is possible that ER stress serves as a downstream signaling pathway for exosomes to regulate osteogenesis and angiogenesis.

## Working principle of ER stress

### Occurrence of ER stress

The ER is the site of protein synthesis and processing [25], the largest intracellular organelle [26]. Proteins tend to enter the ER in an unfolded form, where they begin to fold. However, folding of proteins in the ER is inefficient (< 20%) [27], so protein quality control requires a balance between protein folding and degradation [28]. ER quality control (ERQC) identifies and eliminates misfolded proteins to maintain cellular homeostasis [29]. However, suppression of ERQC by environmental and genetic factors leads to increased protein misfolding [30] and accumulation of misfolded or unfolded proteins in the ER, leading to ER stress [31, 32]. Therefore, ER stress is an important cellular defense mechanism and is vital for maintaining ER homeostasis.

### ER stress signaling pathways

**Fig. 1:**
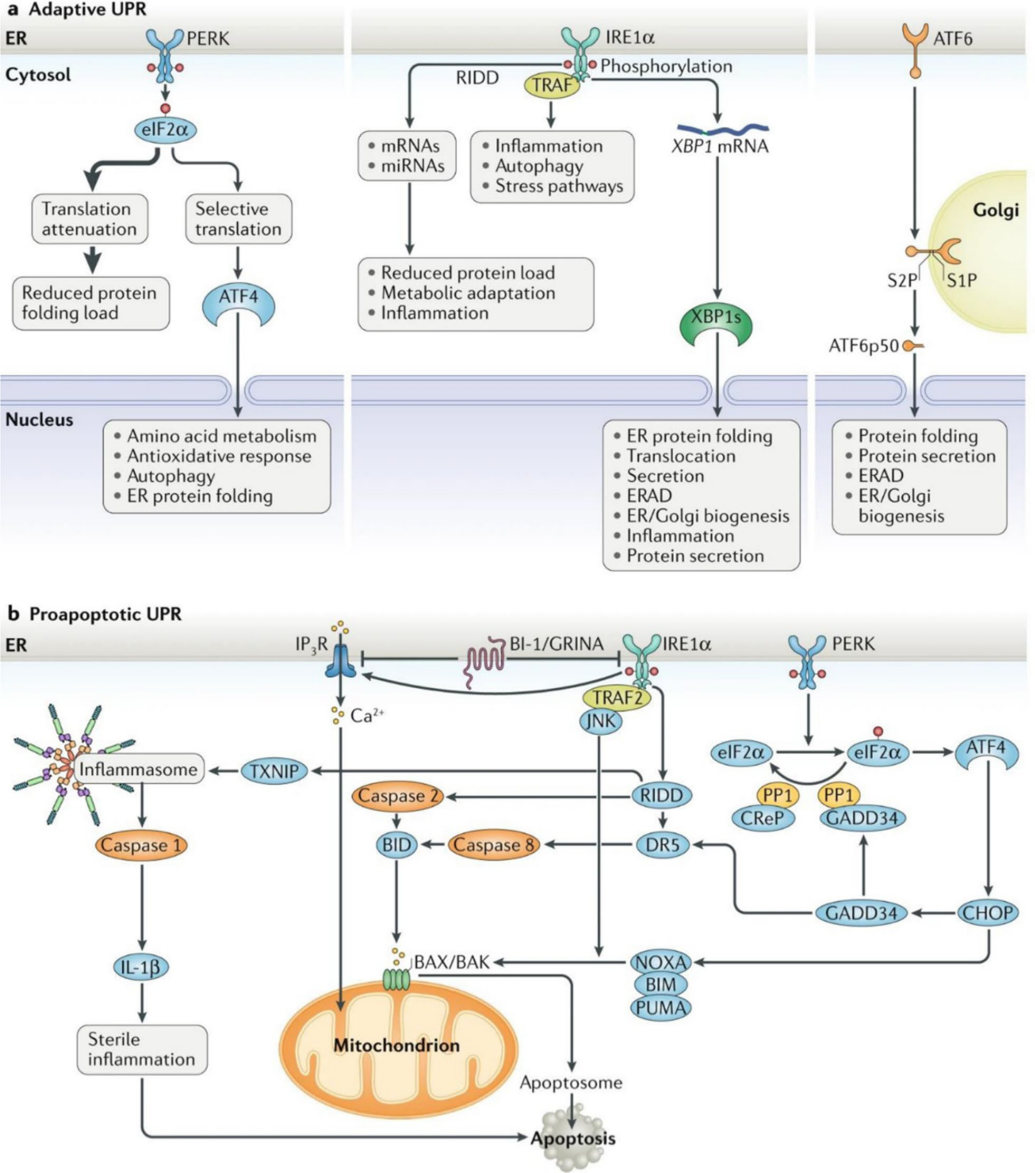
Major UPR pathways initiated in the ER [38]. RIDD: regulated IRE1ɑ-dependent decay; TRAF: tumor necrosis factor receptor associated factor; ERAD: ER-associated protein degradation; PP1: protein phosphatase 1; CreP: constitutive repressor of eIF2ɑ phosphorylation; DR5: death receptor 5; TXNIP: thioredoxin-interacting protein; IP3R: inositol-1,4,5-triphosphate receptor; BI-1: Bax inhibitor-1; GADD34: growth arrest and DNA damage inducible gene 34. Hetz C, Zhang K, Kaufman RJ. Mechanisms, regulation, and functions of the unfolded protein response. Nat Rev Mol Cell Biol. 2020;21(8):421–38. Copyright© The Authors 2020. Published by Springer Ltd

ER stress can be classified as the UPR, ER overload response, and sterol regulatory cascade [33]. UPR occurs when a signal of misfolded ER proteins is transmitted to the nucleus [34]. Ischemia [35, 36], HG [37], and other pathological states activate the ER stress signaling pathway. The UPR is a signal transduction pathway that transmits information about protein folding to the nucleus and cytoplasm to restore ER homeostasis [38] and relieve ER stress [39]. In 1977 glucose-regulated protein (GPR) was discovered [40]. GPR promotes the correct folding of proteins in the ER [41, 42], linking glucose induction to protein misfolding. In 1988, Kozutsumi et al*.* [42] proposed a signaling transduction pathway activated by ER stress. The mammalian UPR pathway was first identified in yeast [43, 44] and is coordinated by three ER transmembrane sensor proteins: protein kinase-R-like ER kinase (PERK), inositol-requiring enzyme 1ɑ (IRE1ɑ), and activating transcription factor 6 (ATF6). It dynamically regulates ER protein folding to maintain ER homeostasis (Fig. 1) [45, 46]. In a non-stressed state, the ER chaperone immunoglobulin heavy-chain binding protein (BiP)/G protein coupled receptor 78 (GPR78) binds to the ER domain to stabilize ATF6 disulfides [38, 47], and PERK and IRE1ɑ bind to BiP and are inactivated [30]. However, in ER stress, BiP dissociates and binds unfolded or misfolded proteins and perform protein folding [48], activating ER receptors [49]. The IRE1ɑ-X-box binding protein (XBP1), PERK-eukaryotic initiation factor 2ɑ (eIF2ɑ), and ATF6 signaling pathways induce the UPR and restore ER stability [50, 51]. IRE1ɑ is the most evolutionarily conserved factor in the UPR [30, 38]. Activated PERK phosphorylates eIF2ɑ, attenuating protein translation to relieve the ER load under stress, and promotes ATF4 translation [30, 45]. The PERK signaling pathway is associated with a series of immune metabolic diseases [52, 53], including tumors [54–56]. ATF6 disulfide is decreased by protein disulfide isomerase (PDI) activity [30], and full-length ATF6 (ATF6p90) monomer increases and is transferred to the Golgi apparatus, where it is cleaved by the site 1 protease (S1P) and site 2 protease (S2P) to release an N-terminal transcriptionally active 50 kDa fragment (ATF6p50) [38, 57]. ATF6p50 is transported to the nucleus to perform functions such as protein folding [38]. ATF6 also maintains the stability of viral proteins [57] and homeostasis in normally developing tissues and organs [58].

### ER stress pathways: a double-edged sword

After the occurrence of ER stress, misfolded or unfolded proteins that accumulate in the ER are eliminated through two primary degradation pathways: ER-associated degradation (ERAD) and autophagy [59]. ERAD is activated in response to ER stress, as it maintains ER homeostasis by eliminating misfolded proteins from the ER and preventing their accumulation [60]. The UPR controls cell fate [17, 19, 20, 61]. A prolonged UPR indicates non-recovery from ER stress, and adaptive output cannot compensate for the pressure in the ER, and the UPR induces apoptosis [20]. Sustained activation of ATF4 in combination with CCAAT-enhancer-binding protein homologous protein (CHOP) induces apoptosis [34]. Thus, the dual role of the PERK-eIF2ɑ axis is vital for coordinating translation and protein balance. There are three main mechanisms (Fig. 1): the IRE1ɑ/ASK1 (apoptosis signal regulating kinase 1)/JNK (c-jun kinase) pathway [62], caspase-12-dependent pathway [63], and growth arrest and DNA damage-inducible 153 (CHOP/GADD153) pathway [64, 65]. CHOP, a key apoptotic factor, upregulates ROS, triggers calcium (Ca^2+^) release, and promotes transcription, constituting a positive-feedback loop that triggers apoptosis [34]. It also downregulates the anti-apoptotic protein B-cell lymphoma 2 (Bcl-2) to induce apoptosis [66]. Despite advances in research on the mechanism of ER stress, the balance between pro-survival and pro-death UPR signals remains unclear, and the full extent of ER stress's role in different stages of disease is yet to be fully elucidated. Future research is necessary to answer these key questions.

## ER stress pathways in orthopedics

Osteoblasts play a critical role in bone formation and reconstruction by synthesizing new collagen. Because osteoblasts secrete a significant amount of extracellular matrix proteins, they are particularly vulnerable to ER stress-induced dysfunction. Targeted ER stress therapy can be used to treat orthopedic diseases. Liu et al. [67] discovered that IL-1β can induce excessive ER stress in chondrocytes, leading to chondrocyte apoptosis and subsequent cartilage degradation, which accelerates the progression of osteoarthritis (OA). Inhibition of ER stress by the IRE1ɑ pathway suppresses chondrocyte apoptosis, thus mitigating the progression of OA [68]. Sim et al. [69] found that the function of ERAD, which is regulated by ER stress, was reduced in patients with OA, leading to the accumulation of misfolded proteins and cartilage loss. Enhanced ERAD activity is necessary for cartilage formation and maintenance. The activation of PERK and ATF4 is involved in the inducing the stress response protein sestrin2 under ER stress after spinal cord injury (SCI) [70]. Inhibiting ER stress through overexpression of sestrin2 promotes functional recovery and neuronal survival, indicating its potential as a therapeutic target for SCI repair. Huang et al. [71] found that inhibition of neuronal apoptosis mediated by ER stress can reduce apoptosis and protects neurons. ER stress has potential to be a new target for treating SCI; Metastasis of osteosarcoma cells can be inhibited by knocking out secretion-associated Ras-related GTPase 1A (SAR1A), a key regulator of ER homeostasis [72]. Moreover, ER stress can induce hypertrophic chondrocyte dysfunction, which may be a potential cause of osteogenesis imperfecta (OI) [73]. Nevertheless, studies have demonstrated that downstream ER stress is necessary to maintain Ol bone integrity to a certain extent. Reducing ER stress alone may not be sufficient to rescue Ol phenotype and may even exacerbate it [74]. Although we have known that ER stress is part of the mechanism of OI disease, how to improve OI bone strength by regulating ER stress remains to be studied.

The repair and regeneration of bone defects caused by trauma, tumor, infection and other factors have been significant clinical challenges. If ER stress can be precisely regulated to an appropriate level through bone tissue engineering or stem cell therapy, it could help in the rapid regeneration of bone tissue. Xiang et al. [75] modulated the expression of osteogenic proteins through the PERK-eIF2ɑ-ATF4 pathway of appropriate ER stress by Ca^2+^ changes mediated by biphasic calcium phosphate, a classic bone void filler. Zheng et al. [76] used the osteogenesis-promoting drug HA15 to target HSPA5 to inhibit excessive ER stress and ultimately promote osteogenesis and angiogenesis in rabbit bone defect models. Future studies can use the involvement of the ER stress pathway in the regulation of osteogenesis and angiogenesis as a starting point through cell experiments, investigate the precise mechanism by which ER stress regulates osteogenesis and angiogenesis, and seek more possible therapeutic targets and interventions in the pathogenesis of bone defect from the level of gene regulation, bringing good news to the majority of patients.

## Effects of ER stress pathways on osteogenesis

### Appropriate ER stress contributes to osteogenic differentiation

Bone morphogenetic proteins (BMPs) are implicated in osteogenic differentiation and ectopic bone formation [77]. BMP2 and BMP9 induce ER stress to promote the differentiation of BMSCs into osteoblasts [78–81]. UPR signaling is an essential regulator of bone development [82, 83].

The three UPR signaling pathways are linked to the promotion of osteogenic differentiation by ER stress. Kazuhisa et al*.* [84] discovered Osterix (Osx), a transcription factor necessary for bone formation. Ten years later, Stavroula [85] identified Osx as a target gene of XBP1, linking ER stress and osteogenesis. The IRE1ɑ-XBP1 signaling pathway promotes not only osteoblast maturation by promoting Osx transcription [86] but also bone regeneration via myostatin mRNA decay [87]. ATF4 is a key transcription factor for osteoblast differentiation and bone formation [88, 89]. Activation of the PERK-eIF2ɑ-ATF4 pathway promotes the expression of genes required for osteogenesis [90] and induces osteogenic differentiation [91, 92] and type I collagen secretion, which are essential for neonatal bone development and osteogenic differentiation [93]. Won-Gu et al*.* [94] showed that BMP2 stimulates osteoblast differentiation by regulating osteocalcin gene expression via the ER stress-activated ATF6 pathway [58]. Although the three UPR signaling pathways are implicated in osteogenesis, the underlying mechanisms are unclear.

### Excessive ER stress induces osteoblast apoptosis

Excessive ER stress inhibits osteogenic differentiation and induces their apoptosis [91, 95, 96], which is an important mechanism of osteoporosis [97]. The effect may be related to the overexpression of CHOP caused by excessive ER stress [98], and there are sex differences in sensitivity to CHOP [99]. Overexpression of CHOP reduces alkaline phosphatase activity and calcified bone nodule formation [100], and initiates osteoblast apoptosis, inhibits bone formation, and induces osteopenia [98, 100].

ER stress-mediated osteoblast apoptosis is driven by an increase in the intracellular Ca^2+^ concentration [101]. An increased intracellular Ca^2+^ disrupts Ca^2+^ homeostasis, leading to Ca^2+^ overload [102] and excessive ER stress103 and inducing osteoblast apoptosis [104, 105]. Furthermore, micronutrients such as cadmium [106], fluorine [107, 108], and iron [109] initiate the ER stress apoptosis pathway by increasing intracellular Ca^2+^. Therefore, controlling intracellular Ca^2+^ has therapeutic potential for micronutrient-induced osteoporosis. We summarize the effects of ER stress inducers on osteogenic differentiation in Table 1.

**Table 1:** ER stress inducers used to modulate osteogenic differentiation

ER stress inducer	Pathway	Stress degree	Up/down	Mechanism	Refs.
TNF-ɑ	JNK	Excessive	Down	Inhibit osteogenic differentiation of BMSCs	[128]
Curcumin	ATF6	Appropriate	Up	Promote osteogenic differentiation of C3H10T1/2 cells	[135]
METTL3	–	Excessive	Down	induce osteoblast apoptosis	[136]
CDs	PERK-eIF2ɑ-ATF4	Appropriate	Up	promote pre-osteoblast differentiation in vitro and bone regeneration in vivo	[137]
PIs	IRE1ɑ-XBP1	Approriate	Up	promote osteogenic differentiation	[138]
Melatonin	PERK-eIF2ɑ-ATF4	Excessive	Down	induce apoptosis in hFOB 1.19 human osteoblastic cells	[139]
AGEs	IRE1ɑ	Excessive	Down	induce apoptosis in osteoblastic MC3T3-E1 and human osteoblastic hFOB 1.9 cells	[140]
TNF-ɑ	PERK	Excessive	Down	inhibit osteogenic differentiation of PDLSCs	[141]
HA15	PERK-eIF2ɑ-ATF4	Appropriate	Up	promote osteogenic differentiation in vitro, and attenuate estrogen deficiency-induced bone loss in vivo	[142]
MNT	PERK-eIF2ɑ-ATF4	Appropriate	Up	Promote osteogenic differentiation of stem cells	[143]
PA	CHOP/Caspase-12 /JNK	Excessive	Down	induce apoptosis in osteoblastic MC3T3-E1 cells	[144]
FTO	A positive feedback loop with p­AMPK	Appropriate	Up	promote osteogenic differentiation of C3H10T1/2 cells	[145]
Metallic wear debris	–	Excessive	Down	induce osteoblast apoptosis	[146]

TNF-ɑ: tumor necrosis factor-ɑ; METTL3: methyltransferase-like 3; CDs: carbon dots; PI: proteasome inhibitor; AGE: advanced glycation end product; PDLSC: periodontal ligament stem cell; MNT: micro-/nano-topography; PA: palmitate; FTO: fat mass and obesity associated

### Regulation of ER stress pathways to interfere with osteogenesis

#### GCs induce osteoblast apoptosis by activating ER stress pathways

A normal concentration of glucose does not activate ER stress [110], but chronic HG induces pancreatic β cells to continuously secrete Ca^2+^ to activate ER stress [37], thus inhibiting osteogenic differentiation in a glucose concentration-dependent manner [110].

Since 1984, glucocorticoids (GCs) have been used for variety of immune-related diseases [111]. However, long-term use of GCs increases the incidence of osteonecrosis, among which osteonecrosis of the femoral head (ONFH) is the most common [112]. Although the mechanism of GC-induced ONFH is unclear, GCs can activate ER stress and promote the production of ROS, thereby inducing apoptosis in osteoblasts [113, 114], particularly in the proximal femur [115]. This may be a mechanism of ONFH.

The PERK-eIF2ɑ-ATF4-CHOP pathway is implicated in GC-induced osteoblast apoptosis [116]. Therefore, controlling this pathway could ameliorate GC-induced osteoblast apoptosis. The plant compound geniposide (GEN) [117], 4-phenylbutyric acid (4-PBA) [117, 118], the PERK phosphorylation inhibitor GSK2656157 [116], and melatonin [119] can block PERK downstream signaling and significantly inhibit ER stress, thereby attenuating GC-induced osteoblast apoptosis. GEN together with the plant compound paeoniflorin activate autophagy in vivo and in vitro, thus suppressing GC-induced apoptosis [115, 120]. 4-PBA downregulates ATF4 and reduces mutant type I collagen [121], whereas salubrinal (inhibitor of eIF2ɑ dephosphorylation) upregulates ATF4 [35, 122]. Both regulate the eIF2ɑ pathway, thereby reducing ER stress to promote osteogenesis. Unfortunately, salubrinal has no effect on osteoblast apoptosis induced by high-dose GC [114].

#### Regulation of ER stress pathways on osteogenesis under inflammatory conditions

Long-term inflammatory responses can affect stem cells' ability to repair [123]. Tumor necrosis factor ɑ (TNF-ɑ)-induced inflammation has been reported to inhibit osteogenic differentiation of BMSCs [124], possibly because ER stress-activated nuclear factor κB (NF-κB) translocates into the nucleus to promote the transcription of other pro-inflammatory cytokines [125] and osteolysis [126]. Xue et al. [96] found for the first time that long-term chronic inflammation reduces the expression of lysine acetyltransferase 6B (KAT6B, also known as MORF), which leads to continuous activation of PERK signaling pathway downstream of ER stress, and reduces the osteogenic differentiation ability of periodontal ligament stem cells (PDLSCs).

Subsequently, Li et al. [127] used low-intensity pulse ultrasound to up-regulate the osteogenic effect of PDLSCs under inflammatory conditions through UPR. Zhao et al. [128] demonstrated that JNK pathway activated by ER stress mediates TNF-ɑ-induced inflammation in BMSCs. These studies have confirmed that inhibiting ER stress can effectively reduce inflammatory response and enhance the osteogenic differentiation ability of stem cells, which may provide new insights for improving stem cell osteogenic differentiation and treating inflammatory bone diseases such as osteoporosis, so that inhibiting ER stress under inflammatory conditions to promote osteogenesis has great potential.

#### Exosomes regulate osteogenesis by activating ER stress pathways

**Fig. 2:**
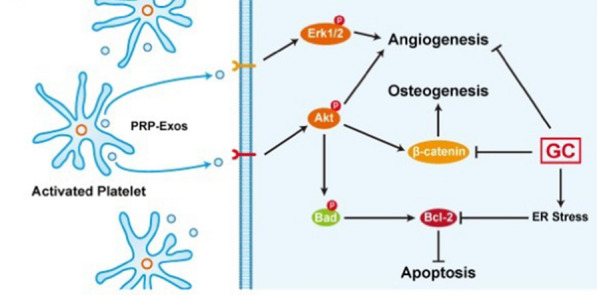
PRP-Exos rescued cells from GC-induced apoptosis via the Akt/Bcl-2 pathway [132]. Tao SC, Yuan T, Rui BY, Zhu ZZ, Guo SC, Zhang CQ. Exosomes derived from human platelet-rich plasma prevent apoptosis induced by glucocorticoid-associated endoplasmic reticulum stress in rat osteonecrosis of the femoral head via the Akt/Bad/Bcl-2 signal pathway. Theranostics. 2017;7(3):733–50. Copyright© The Authors 2017. Published by Ivyspring International Publisher

Studies have shown that miRNA from exosomes of different cellular origins can enter recipient cells and then regulate the expression of genes associated with osteogenesis at the translational level to regulate osteogenesis [129]. We have also reviewed the use of exosome-derived non-coding RNAs for osteogenesis before [130]. However, whether exosomes promote osteogenesis by regulating ER stress is unclear. Platelet-rich plasma (PRP) has been widely used in clinical repair of bone and soft tissue injuries. Recent studies have shown that PRP contains a large number of extracellular vesicles [131]. Tao et al. [132] found that PRP-derived exosomes (PRP-Exos) binds to related receptors, promotes Akt phosphorylation, activates β-catenin to promote osteogenesis, and activates Bcl-2 to inhibit GC-induced apoptosis and ER stress (Fig. 2). Exosomes show great potential in PRP repair tissues, which is closely related to downstream ER stress pathways. Wang et al*.* [133] reported that miR-485–5p modified exosomes inhibit ER stress and alleviate chondrocyte apoptosis for the treatment of OA. Liao et al. [134] demonstrated that BMSCs-derived exosomes (BMSCs-Exos) can improve the apoptosis of nucleus pulposus cells induced by ER stress. Can BMSCs-Exos attenuate osteoblast apoptosis by inhibiting excessive ER stress? This may be a new mechanism of exosome promoting osteogenesis, which needs to be verified by future experiments.

## Effects of ER stress pathways on angiogenesis

### Appropriate ER stress contributes to angiogenesis

ER stress promotes the differentiation of monocytes into ECs, leading to angiogenesis [147, 148], suggesting that ER stress can promote angiogenesis. The angiogenic effect of ER stress is mediated by regulation of angiogenic factors by the UPR. Appropriate ER stress triggers the production of angiogenic factors [149, 150]; however, the mechanism is unclear.

Three UPR signaling pathways bind to regulatory regions of VEGFA, and jointly drive VEGFA transcription [151, 152]. ER stress initiates angiogenesis signaling via UPR-mediated upregulation of VEGFA [153, 154]. The inducible ER chaperone oxygen-regulated protein 150 (OPR150) promotes the expression of VEGFA in pathological conditions and is a potential target for regulating angiogenesis [155]. Under ER stress, the IRE1ɑ-XBP1 pathway promotes tumor angiogenesis [156]– [158], the PERK-ATF4 pathway promotes bone angiogenesis [153, 159], and the ATF6 pathway promotes embryonic angiogenesis [160], by upregulating VEGFA. Binet et al*.* [161] reported a pro-angiogenic role for the UPR in diseases characterized by pathological vascular abnormalities. Therefore, targeted regulation of angiogenesis through the UPR has therapeutic potential for vascular necrotizing diseases. VEGFA can spontaneously increase in acute myocardial ischemia, inducing intracellular Ca^2+^ overload and activating ER stress in a positive-feedback loop [162]. Excessive ER stress induces BMSCs apoptosis [128], and VEGFA stimulates the differentiation of BMSCs into ECs, thus protecting BMSCs and promoting angiogenesis [163]. Increased spontaneous VEGFA production also promotes compensatory angiogenesis through the ROS-ER stress-autophagy axis [162].

The UPR also regulates vascular growth factors. For example, ER stress promotes angiogenesis by upregulating interleukin 8 (IL-8) [164], FGF2 [36], placental growth factor (PIGF) [165], and granulocyte–macrophage colony stimulating factor (GM-CSF) [166] via different transcriptional mechanisms. However, pentraxin 3 (PTX3) has a high affinity for FGF2 and can inhibit its angiogenesis [167, 168], but Ma et al*.* [169] found that the ATF4 pathway activates SMAD-specific E3 ubiquitin ligase 2 and leads to PTX3 degradation, thus promoting angiogenesis. Philippe et al*.* [36] demonstrated that the PERK pathway activates the translation of dependent internal ribosome entry site (IRES), thereby promoting the expression of the angiogenic factors VEGFA and FGF2. These studies have suggested potential therapeutic targets for ischemia in vascular necrotizing diseases.

### Excessive ER stress impairs angiogenesis

Excessive ER stress impairs angiogenesis not only by reducing the transcription of pro-angiogenetic growth factors such as VEGFA [170– 172] and PIGF [173] but also by activating negative angiogenic regulators such as delta-like 4 (DLL4) IRES [174]. Excessive ER stress can induce apoptosis of ECs [175–178], thus suppressing their angiogenesis [171]. Maamoun et al. [179] showed that ER stress causes EC dysfunction, suggesting that targeting ER stress could promote angiogenesis (Table 2). The ER stress-mediated decreased expression of angiogenic genes is related to age [180].

**Table 2:** ER stress inhibitors used to promote angiogenesis

ER stress inhibitor	Pathway	Animal model	Mechanism	Refs.
GB	CHOP, GPR78, caspase-12	Rat	Promote perforator flap angiogenesis	[210]
Quercetin	ATF6/GPR78	N/A	Protect HBMECs and promote angiogenesis	[211]
Vitamin D	GPR78, JNK1, eIF2ɑ, XBP-1	N/A	Protect HUVECs and promote angiogenesis	[212]
PTP1B inhibition	PI3K/Akt	N/A	Protect HUVECs, activate eNOS and promote angiogenesis	[171]
SFN	ATF6/GPR78	Chick	Promote embryo angiogenesis	[213]
Naringin	GPR78, CHOP, caspase-12, Cyt.c	Rat	Protect VECs and promote angiogenesis	[214]
Salubrinal	eIF2ɑ-ATF4-GPR78	Rat	Promote HUVESs, upregulate VEGFA and promote angiogenesis	[35]
HO-1	BiP, PERK-eIF2ɑ-ATF4	N/A	Alleviate HG-induced HUVECs apoptosis and promote angiogenesis	[172]
PCB2	PERK, IRE1ɑ and ATF6	Mouse	Alleviate HG-induced ECs dysfunction and promote angiogenesis	[192]
streptozotocin	CHOP	Mouse	Alleviate HG-induced APCs dysfunction and promote vascular repair	[193]
GSK2656157	PERK	Mouse	Alleviate GCs-induced ECs apoptosis and promote angiogenesis	[116]

GB: Ginkgolide B; N/A: no animal; HBMECs: human brain microvascular endothelial cells; HUVECs: human umbilical endothelial cells; PTP1B: protein tyrosine phosphatase 1B; eNOS: endothelial nitric oxide synthase; SFN: sulforaphane; Cyt.c: cytochrome c; VECs: vascular endothelial cells; HO-1: Hemeoxygenase-1; PCB2: procyanidin B2; APCs: angiogenic progenitor cells

The anti-angiogenic effect of ER stress also has benefits, such as inhibiting cancer progression [181]. ER stress can induce the expression of miR-153, which inhibits angiogenesis by two mechanisms, suggesting a novel therapeutic strategy for breast cancer [182].

### Regulation of ER stress pathways to interfere with angiogenesis

#### HG impairs angiogenesis by activating ER stress pathways

In diabetic retinopathy (DR), HG damages normal blood vessels and causes abnormal neovascularization [183, 184]. ER stress is closely related to retinal angiogenesis [185]. Wang et al*.* [186] showed that regulation of ER stress can inhibit abnormal neovascularization. However, whether damaged normal blood vessels can be restored by regulating ER stress is unknown.

HG rapidly activates ER stress in ECs [187] and angiogenic progenitor cells (APCs) [188], leading to microvascular EC dysfunction and impair angiogenesis [189]. ECs have a greater apoptotic effect under GC induction than do other cells [190, 191]. Gao et al*.* [116] demonstrated that GCs induce EC apoptosis by activating ER stress, leading to microvascular damage. Alleviating the ER stress induced by HG can counteract HG-induced EC apoptosis [172], thus restoring angiogenesis [192] and enhancing vascular repair by circulating angiogenic cells (CACs) [193] (Table 2). Inhibition of ER stress can prevent vascular damage by upregulating pro-angiogenic factors and downregulating anti-angiogenic factors [194]. Wang et al*.* [195] found that an atypical UPR pathway mediated by IRE1ɑ regulates miRs, thereby protecting the pro-angiogenic growth factor angiopoietin 1 (ANGPT1) from miR attack under HG conditions and promoting bone marrow–derived progenitor cell (BMPC) angiogenesis. Therefore, targeting ER stress is the key to reversing HG-induced vascular injury.

#### Regulation of ER stress pathways on angiogenesis under inflammatory conditions

In recent years, ER stress pathways secondary to inflammation have become new targets for intracellular therapy. ER stress can induce nucleotide-binding domain and leucine-rich repeat containing (NLRP3) inflammasome through PERK and IRElα pathways, regulate the release of inflammatory cytokines, and trigger inflammatory response [196]. Wang et al. [197] demonstrated that there is a positive feedback loop between interleukin-17A (IL-17A) and ER stress, and that inhibition of ER stress or IL-17A can reduce the neovascularization area of DR. At present, inhibition of ER stress can alleviate inflammation and inhibit angiogenesis, which has been proved in both cell and animal experiments [186, 198]. Although ER stress pathway shows great potential in anti-inflammatory and anti-vascular therapy, more in-depth mechanism studies are needed before clinical trials.

#### Exosomes regulate angiogenesis by activating ER stress pathways

Exosomes promote angiogenesis by inducing the regeneration of damaged blood vessels by inhibiting EC apoptosis and promoting their angiogenic activity [199–202]. Tumor cell-derived exosomes deliver miR-25-3p to ECs, thereby disrupting ECs integrity, increasing vascular permeability, and promoting angiogenesis, thereby promoting tumor metastasis [203]. Based on the role of ER stress in numerous pathological conditions, whether exosomes promote angiogenesis by regulating ER stress is a topic of interest. Tao et al*.* [132] have found that PRP-Exos activates the Akt pathway under ER stress, releasing multiple growth factors and promoting angiogenesis (Fig. 2).

Exosomes have a dual regulatory effect on angiogenesis. Angiogenesis can be inhibited by exosomes. For example, exosomal circular RNAs (circRNAs) act as signal carriers to trigger EC dysfunction [204], exosomes can enhance the inhibitory effect of the anti-angiogenic peptide KV11 on pathological retinal angiogenesis [205], and circulating exosomal miR-20b-5p is transferred to vascular ECs to inhibit the regeneration of diabetic damaged blood vessels [206]. Wang et al*.* showed that ER-stressed HN4 cell-derived exosomes modified by miR-424–5p inhibit angiogenesis by HUVECs [207].

Exosomes from different sources have different regulatory effects on angiogenesis under ER stress. Until now, studies on exosomes promoting angiogenesis by activating ER stress have focused on exosomes of tumor cell origin. Lin et al. [208] demonstrated that after knocking down PERK in HUVEC, HeLa cell-derived exosomes can significantly improve HUVEC proliferation. We know that BMSCs-Exos have great potential in promoting angiogenesis [209], but whether ER stress may be a downstream pathway and whether we can enhance the ability of BMSCs-Exos to promote angiogenesis by regulating ER stress needs to be demonstrated in future studies.

## Potential interventions related to ER stress pathways

Because the ER controls protein synthesis and degradation, ER stress is used clinically to restore myogenic differentiation to treat uremic sarcopenia [215]. Moreover, clinical trials by Bella et al. [216] suggested that ER stress may play a key role in the pathogenesis of amyotrophic lateral sclerosis by altering the regulation of protein balance, and that molecules acting on functional control of the UPR pathway may be beneficial in slowing disease progression, but subgroup analyses were not performed in this study. Therefore, this effect on targeting ER stress is considered exploratory. Besides, drugs targeting the IRE1ɑ-XBP1 pathway can inhibit vascular smooth muscle apoptosis, thereby alleviating aortic dissection [217]. Dexmedetomidine pretreatment can effectively protect myocardial ischemia–reperfusion-induced acute kidney injury by inhibiting ER stress [218].

Regulation of ER-related signaling pathways is most commonly used in the treatment of tumor diseases. ER stress is an essential intermediate targeting pathway in tumor therapy. Activation of ER stress can increase the cytotoxicity of photodynamic therapy to tumor cells [219]. Chemotherapy can increase tumor (sarcoma and gastric cancer) sensitivity by activating ER stress [220, 221]. Use of some chemotherapy drugs is limited by their toxicity. However, drugs that inhibit ER stress have reduced toxicity, and can be used in chemotherapy for cancer [222, 223]. Basic research by Varone et al. [224] showed that ISRIB (a small molecule that inhibits the action of phosphorylated eIF2ɑ) increases ER protein load, reactivates protein synthesis in damaged protein homeostasis, and ultimately promotes tumor cytotoxicity. ISRIB offers a new treatment option that can effectively inhibit tumor progression in conditions with impaired protein balance.

Although the mechanism of ER stress has been relatively clear, the current research on the intervention effect of ER stress in many diseases such as different types of diabetes and its complications is far from enough. Regulating a key signaling pathway node in the complex process of ER stress to affect the occurrence and development of diseases is an important target for drug therapy exploration, which has important clinical guiding value and practical significance. Further large-scale and long-term studies are needed to confirm the clinical benefits of this new pharmacological protocol, which may provide a promising therapeutic approach for targeted therapies for a number of diseases in the clinic.

## Conclusion and perspective

In regenerative medicine, bone defects can be improved by promoting angiogenesis and osteogenesis. Our research has focused on inducing the regeneration of dead blood vessels and bone. ER stress is involved in many diseases. ER stress is a double-edged sword; its activation can promote cell generation, but excessive activation can induce apoptosis. ER stress plays a dual role in osteogenesis and angiogenesis, and thereby determines cell fate. Here we systematically reviewed the effect of ER stress on osteogenesis and angiogenesis. ER stress can be activated in pathological conditions such as HG and inflammation, or by inducers, and is inactivated by inhibitors. Therefore, regulation of ER stress has potential as a therapeutic target to promote osteogenesis and angiogenesis. Although regulating ER stress stimulates osteogenesis and angiogenesis, the mechanism is unclear. Efforts should focus on unraveling the mechanisms underlying the roles of ER stress in osteogenesis and angiogenesis.

Acellular therapy, such as exosome-mediated regulation of ER stress, is a focus of research. BMSCs-Exos have great potential for osteogenesis and angiogenesis, and we propose to hypothesize that ER stress can act as a downstream pathway for their regulation. Our future studies will further clarify the mechanism by which BMSCs-Exos promote angiogenesis and bone regeneration by regulating ER stress. Further research on the mechanism of ER stress regulating osteogenesis and angiogenesis will be helpful for the repair of bone defects.

## Data Availability

Not applicable.

## Abbreviations

ER: Endoplasmic reticulum
UPR: Unfolded protein response
HG: High glucose
ECs: Endothelial cells
VEGF: Vascular endothelial growth factor
FGF: Fibroblast growth factor
ERQC: ER quality control
GPR: Glucose-regulated protein
PERK: Protein kinase-R-like ER kinase
IRE1ɑ: Inositol-requiring enzyme 1ɑ
ATF6: Activating transcription factor 6
BiP: Immunoglobulin heavy-chain binding protein
GPR78: G protein coupled receptor 78
XBP1: X-box binding protein
eIF2ɑ: Eukaryotic initiation factor 2ɑ
PDI: Protein disulfide isomerase
ATF6p90: Full-length ATF6
S1P: Site 1 protease
S2P: Site 2 protease
ATF6p50: 50 KDa fragment
CHOP: CCAAT-enhancer-binding protein homologus protein
ASK1: Apoptosis signal regulating kinase 1
JNK: C-jun kinase
GADD153: Growth arrest and DNA damage-inducible 153
ROS: Reactive oxygen species
Ca2 +: Calcium
Bcl-2: B-cell lymphoma 2
RIDD: Regulated IRE1ɑ-dependent decay
TRAF: Tumor necrosis factor receptor associated factor
ERAD: ER-associated protein degradation
PP1: Protein phosphatase 1
CreP: Constitutive repressor of eIF2ɑ phosphorylation
DR5: Death receptor 5
TXNIP: Thioredoxin-interacting protein
IP3R: Inositol-1,4,5-triphosphate receptor
BI-1: Bax inhibitor-1
GADD34: Growth arrest and DNA damage inducible gene 34
OA: Osteoarthritis
IDD: Intervertebral disc degeneration
SCI: Spinal cord injury
SAR1A: Secretion-associated Ras-related GTPase 1A
OI: Osteogenesis imperfecta
BMPs: Bone morphogenetic proteins
BMSCs: Bone marrow mesenchymal stem cells
Osx: Osterix
GCs: Glucocorticoids
ONFH: Osteonecrosis of the femoral head
GEN: Geniposide
4-PBA: 4-Phenylbutyric acid
PRP-Exos: Exosomes derived from platelet-rich plasma
TNF-ɑ: Tumor necrosis factor-ɑ
METTL3: Methyltransferase-like 3
CDs: Carbon dots
PI: Proteasome inhibitor
AGE: Advanced glycation end product
PDLSC: Periodontal ligament stem cell
MNT: Micro-/nano-topography
PA: Palmitate
FTO: Fat mass and obesity associated
OPR150: Oxygen-regulated protein 150
IL-8: Interleukin 8
